# Relationships between Serotonin Transporter Binding in the Raphe Nuclei, Basal Ganglia, and Hippocampus with Clinical Symptoms in Cervical Dystonia: A [^11^C]DASB Positron Emission Tomography Study

**DOI:** 10.3389/fneur.2018.00088

**Published:** 2018-02-28

**Authors:** Marenka Smit, David Vállez García, Bauke M. de Jong, Evelien Zoons, Jan Booij, Rudi A. Dierckx, Antoon T. Willemsen, Erik F. de Vries, Anna L. Bartels, Marina A. Tijssen

**Affiliations:** ^1^Department of Neurology, University Medical Center Groningen (UMCG), University of Groningen, Groningen, Netherlands; ^2^Department of Nuclear Medicine and Molecular Imaging, University Medical Center Groningen (UMCG), University of Groningen, Groningen, Netherlands; ^3^Department of Neurology, Academic Medical Center (AMC), University of Amsterdam, Amsterdam, Netherlands; ^4^Department of Nuclear Medicine and Molecular Imaging, Academic Medical Center (AMC), University of Amsterdam, Amsterdam, Netherlands; ^5^Department of Neurology, Ommelander Hospital Group, Groningen, Netherlands

**Keywords:** dystonia, serotonin, positron emission tomography, DASB, cervical dystonia

## Abstract

**Purpose:**

Alterations of the central serotonergic system have been implicated in the pathophysiology of dystonia. In this molecular imaging study, we assessed whether altered presynaptic serotonin transporter (SERT) binding contributes to the pathophysiology of cervical dystonia (CD), concerning both motor and non-motor symptoms (NMS).

**Methods:**

We assessed the non-displaceable binding potential (BP_ND_) using the selective SERT tracer [^11^C]DASB and positron emission tomography (PET) in 14 CD patients and 12 age- and gender-matched controls. Severity of motor symptoms was scored using the Toronto Western Spasmodic Torticollis Rating Scale and Clinical Global Impression jerks/tremor scale. NMS for depressive symptoms, anxiety, fatigue, and sleep disturbances were assessed with quantitative rating scales. The relationship between SERT binding and clinical patient characteristics was analyzed with the Spearman’s rho test and multiple regression.

**Results:**

When comparing the CD patients with controls, no significant differences in BP_ND_ were found. Higher BP_ND_ in the dorsal raphe nucleus was statistically significantly correlated (*p* < 0.001) with motor symptom severity (*r*_s_ = 0.65), pain (*r*_s_ = 0.73), and sleep disturbances (*r*_s_ = 0.73), with motor symptom severity being the most important predictor of SERT binding. Furthermore, fatigue was negatively associated with the BP_ND_ in the medial raphe nucleus (*r*_s_ = −0.61, *p* = 0.045), and sleep disorders were positively associated with the BP_ND_ in the caudate nucleus (*r*_s_ = 0.58, *p* = 0.03) and the hippocampus (*r*_s_ = 0.56, *p* = 0.02).

**Conclusion:**

Motor symptoms, as well as pain, sleep disturbances, and fatigue in CD showed a significant relationship with SERT binding in the raphe nuclei. Moreover, fatigue showed a significant relationship with the medial raphe nucleus and sleep disorders with the caudate nucleus and hippocampus. These findings suggest that an altered serotonergic signaling in different brain areas in CD is related to different motor as well as NMS, which will further stimulate research on the role of serotonin in the pathogenesis of dystonia.

## Introduction

Cervical dystonia (CD) is a movement disorder characterized by involuntary abnormal muscle contractions of the neck. Non-motor symptoms (NMS) like depressive symptoms, pain, and fatigue are prevalent in up to 95%of CD patients (1).

Although the pathogenesis of CD is largely unknown, alterations of the serotonergic system have been suggested to play a major role in both motor symptoms and NMS in dystonia [for review, see Ref. (2)]. A dense projection of fibers from serotonergic neurons, located within the raphe nuclei in the brainstem, arises to important structures of the basal ganglia motor control system, such as the striatum and internal globus pallidus (GPi). The GPi plays a key role in the network underlying the pathophysiology of dystonia (3, 4), a contribution supported by the therapeutic effect of GPi deep brain stimulation for dystonia (3).

Further evidence that alterations of the serotonergic system may play a role in the pathogenesis of dystonia, including CD, can be inferred from case reports describing dystonia induced by selective serotonin reuptake inhibitors. Also, in children with dopa-responsive dystonia as well as in adults with idiopathic adult-onset dystonia decreased levels of serotonin metabolites in cerebrospinal fluid have been reported (5).

Positron emission tomography (PET) provides a unique opportunity to study the serotonin transporter (SERT), an important presynaptic marker of serotonergic functioning *in vivo*. The PET radioligand [^11^C]DASB is a well-validated selective radiotracer to measure SERT binding in the human brain. In Parkinson’s disease, [^11^C]DASB PET studies have shown that aberrations of SERT in different brain areas are related to both motor and NMS, including tremor severity (6), dyskinesia (7), fatigue (8), and depressive symptoms (9).

In this study, we examined SERT binding in CD patients and matched controls using [^11^C]DASB PET imaging and corrected for the SERT gene-linked polymorphism. Based on previous findings, we hypothesized that dysfunction of the serotonergic system is a shared pathophysiological pathway for motor- and NMS in CD patients.

## Materials and Methods

### Subjects

This case-control study included 14 patients with a clinically diagnosed idiopathic CD and 14 age- and sex-matched controls.

Exclusion criteria included the onset of CD before the age of 18 years, and severe tremor and/or jerks preventing accurate brain imaging due to movement artifacts. Exclusion criteria for all subjects were other relevant neurological comorbidity and the current use of serotonergic medication or antidepressants. Written informed consent was obtained from all participants in accordance with the declaration of Helsinki and the study was approved by the local ethics committee of the University Medical Center Groningen (2014/034). The study was not registered in a public trial registry.

### Clinical Measures

Motor assessment was performed using a systematic video protocol. If patients were treated with botulinum toxin (*n* = 12), the clinical assessment was performed within two weeks prior to or one week after botulinum toxin treatment in order to obtain the least influenced motor score. CD motor symptom severity and related pain were scored with the Toronto Western Spasmodic Torticollis Rating Scale (TWSTRS) (10); severity of jerks and tremor were scored using the Clinical Global Impression Scale: CGI-S jerks-tremor (11). Motor function was independently scored by two experts (MS and VH) and the average score was used in the statistical analysis as there was good inter-observer agreement (intraclass correlation coefficients > 0.70, two-way mixed, absolute agreement).

Non-motor symptoms, including depressive symptoms, anxiety, fatigue, and sleep disturbances, were assessed using the Beck Depression Inventory (BDI), the Beck Anxiety Inventory (BAI), the Fatigue Severity Scale (FSS), and the Pittsburgh Sleep Quality Index (PSQI) (2).

### Genetics

The SERT gene-linked polymorphic region (5-HTTLPR), encoded by the *SLC6A4* gene, is an important regulator of the number of expressed SERTs. Three different alleles in this polymorphic region have been associated with changes in transcriptional activity. Functionally, the S and L_G_ allele induce low-transcriptional activity, while the L_A_ allele induces high-transcriptional activity (12). We classified 5-HTTLPR status as L_A_/L_A_ genotype or L_G_/S-allele carrier; details of the methodology are explained in the Supplementary Material.

### PET and MRI Data Acquisition

Positron emission tomography imaging was performed either with a Biograph 40-mCT or 64-mCT (Siemens Healthcare, USA). Head movement was minimized with a head-restraining band. After a low-dose CT for attenuation and scatter correction, a dynamic 60-min data acquisition scan (23 frames: 7 s × 10 s, 2 s × 30 s, 3 min × 1 min, 2 min × 2 min, 2 min × 3 min, 5 min × 5 min, and 2 min × 10 min) was started simultaneously to an intravenous bolus injection of [^11^C]-3-amino-4-(2-dimethylaminomethyl-phenylsulfanyl)benzonitrile ([^11^C]DASB) (379 ± 44 MBq). Synthesis of [^11^C]DASB was performed by methylation of *N*-methyl-2-(2-amino-4-cyanophenylthio)-benzylamine, and the details have been reported elsewhere (13).

Individual axial 3D T1-weighted gradient-echo images (3T Intera, Philips, the Netherlands) of the brain were acquired from all participants. MR images were visually examined (AB) and no significant structural lesions were detected.

### Image Reconstruction and Preprocessing

The list-mode data from the PET scans were reconstructed using the 3D OSEM algorithm (3 iterations and 24 subsets), point spread function correction, and time-of-flight, resulting in a matrix of 400 × 400 × 111 of isotropic 2-mm voxels, smoothed with 2-mm filter at full width at half maximum (FWHM).

Image processing and pharmacokinetic analysis were performed with PMOD v3.7 software (PMOD Technologies Ltd., Switzerland). Motion correction was applied to the PET images (frames 9–23, using frame 15 as reference) to account for the movement of the subject during the scan. Then, the summed image (frames 13–23) was used for rigid matching registration of the individual PET to the individual MRI. A three-tissue probability map normalization of the individual MRI into the Montreal Neurological Institute (MNI) standard space was calculated and subsequently applied to the corresponding PET image (14).

Predefined volumes of interest (VOIs) were transformed back into individual space, based on the Hammers atlas (15) and limited to the gray-matter tissue of cortical regions (>30% gray-matter probability based on individual segmentations). In addition, VOIs for the dorsal raphe nucleus (dRN) and the median raphe nucleus (mRN) were manually defined based on their MNI coordinates (atlas based, i.e., dRNa and mRNa) (16). Moreover, driven by local signal intensity, extra definitions of dRN and mRN VOIs were delineated by selecting 80% of the voxels with the highest uptake within a sphere (diameter of 8 mm for the dRN and 6 mm for mRN) manually located in these regions, according to visual inspection of the PET image (average of frames 20–23) (subject based, i.e., dRNs and mRNs).

After spatial registration of the images, pharmacokinetic modeling was performed to obtain the [^11^C]DASB non-displaceable binding potential (BP_ND_) (17), using the simplified reference tissue model 2 (SRTM2) (18) with the cerebellum (excluding vermis) as the reference region. BP_ND_ values were obtained from aligned PET images in the individual space for the VOI-based analysis, and in the MNI space for the voxel-based analysis. A Gaussian kernel of 6-mm FWHM was applied to the PET image before voxel-based pharmacokinetic modeling.

### Statistical Analysis

Statistical analysis was performed using SPSS Statistics 22 (IBM SPSS Statistics, USA). Demographic and clinical data and the values obtained from the VOI-based modeling were compared between groups using the Pearson’s χ^2^ test/Fisher’s exact test or the Mann–Whitney *U* test. Differences were considered statistically significant at <5% probability (*p* < 0.05) of equality. In the case of significant differences between groups, we additionally performed correction for multiple comparisons. Additionally, effect sizes were calculated using the Cohen’s *d* test, which were interpreted as *d* > 0.1: small effect; *d* > 0.3: medium effect; and *d* > 0.5: large effect (19).

For statistical comparison of the BP_ND_ parametric images, a two-sample *t*-test was performed in SPM12 (Wellcome Trust Centre for Neuroimaging, UK) between dystonia patients and controls. For interpretation of the results, T-maps data were interrogated at *p* = 0.005 (uncorrected) and only clusters with *p* < 0.05 corrected for family wise error were considered significant.

Correlation analysis between the BP_ND_ obtained from the VOI-based modeling and clinical characteristics was performed using the Spearman’s rho test. With multiple regression analysis, we then determined the influence of clinical variables with a *p*-value <0.05 in the univariate analysis on the BP_ND_. Assumptions of the linear regression and multicollinearity were checked.

## Results

### Clinical Characteristics

Fourteen patients (mean age 56 years, range 46–70) and 14 controls (mean age 54 years, range 39–70) were included in the study. Two controls, however, were excluded from the analysis. One healthy control prematurely stopped the scanning procedure. The other subject was excluded due to subcutaneous infusion of the tracer. After excluding these two controls from the analysis, the two groups under study were still age- and gender matched (Table 1).

**Table 1:** Demographic and clinical characteristics.

	CD (*n* = 14)	Controls (*n* = 12)	Maximum value	Clinical relevant	*p*-Value
Age	56 ± 9 years	53 ± 8 years			ns
Female	12 (86%)	10 (83%)			ns
Dystonia duration	12 ± 13 years				
TWSTRS					
– Motor severity	17.5 ± 5.7		35		
– Disability	11.6 ± 5.7		30		
– Pain	7.2 ± 6.4		20		
– Total	36.4 ± 15.9		85		
CGI-S tremor/jerks	1.9 ± 0.8		7		
BDI	12.6 ± 6.6	3.6 ± 3.9	63	≥10	0.01
BAI	8.9 ± 5.6	3.9 ± 3.6	63	≥10	<0.01
FSS	38.8 ± 14.3	22.9 ± 6.6	63	≥36	<0.01
PSQI	8.3 ± 3.8	5.3 ± 4.3	21	≥5	ns
L_A_/L_A_ genotype	3 (21%)	5 (42%)			ns
Injected dose [^11^C]DASB (MBq)	387 ± 33.5	371 ± 53			ns

*Demographic and clinical characteristics of CD patients and controls, including the maximum values of the different motor and non-motor scales and clinically relevant values. Data shown as mean ± SD or number (%)*.

*CD, cervical dystonia, Y, years; TWSTRS, Toronto Western Spasmodic Torticollis Rating Scale; CGI-S, Clinical Global Impression Scale; BDI, Beck Depression Inventory; BAI, Beck Anxiety Inventory; FSS, Fatigue Severity Scale; PSQI, Pittsburgh Sleep Quality Index*.

The patients had a median dystonia duration of 9 years (range 1–52) years. The total TWSTRS score was 36.4 (SD ± 15.9) and the CGI-S jerks/tremor score 1.9 (SD ± 0.8). All patients had a combination of rotation and laterocollis, with some patients also having antero- or retrocollis, lateral- or sagittal shift, or very mild tremor. Most of the measured NMS scores were statistically significantly higher in the CD patients as compared with the controls, with values for depressive symptoms (BDI) of 12.6 (SD ± 6.6) vs. 3.6 (SD ± 3.9) (*p* < 0.01), anxiety (BAI) 8.9 (SD ± 5.6) vs. 3.9 (SD ± 3.6) (*p* = 0.01), and fatigue (FSS) 38.8 (SD ± 14.3) vs. 22.9 (SD ± 6.6) (*p* < 0.01). Sleep disturbances (PSQI) were also more severe in CD patients, but the difference did not reach statistical significance: 8.3 (SD ± 3.8) vs. 5.3 (SD ± 4.3) (*p* = 0.16).

The prevalence of the L_A_/L_A_ genotype was not significantly different in patients and controls (42 vs. 21%, *p* = 0.40).

### Volumes of Interest-Based Analysis of [^11^C]DASB Binding

**Figure 1:**
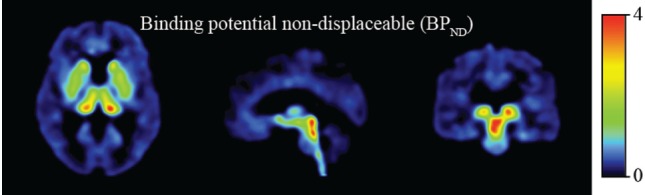
Binding potential non-displaceable (BP_ND_).

The distribution of [^11^C]DASB in control subjects revealed strong binding in the dorsal midbrain, thalamus, and striatum (Figure 1).

No statistically significant differences in the BP_ND_ of SERT between CD patients and controls were detected with the VOI-based analysis (Table 2). Neither were significant asymmetries detected between the left and right VOIs in any of the groups (data not shown).

**Table 2:** BP_ND_ in CD patients and controls in volumes of interest (VOIs).

	CD (*n* = 14)	Controls (*n* = 12)
Frontal cortex	0.33 ± 0.07	0.36 ± 0.06
Anterior cingulate cortex	0.43 ± 0.08	0.47 ± 0.09
Thalamus	1.24 ± 0.24	1.31 ± 0.22
Caudate nucleus	0.90 ± 0.37	0.89 ± 0.33
Pallidum	1.39 ± 0.30	1.51 ± 0.27
Putamen	1.57 ± 0.29	1.73 ± 0.27
*S*. Nigra	1.96 ± 0.66	1.82 ± 0.21
Temporal lobe	0.22 ± 0.06	0.22 ± 0.05
Hippocampus	0.49 ± 0.08	0.54 ± 0.09
Amygdala	1.58 ± 0.38	1.65 ± 0.34
Insula	0.62 ± 0.11	0.68 ± 0.11
dRN		
– dRNa	5.29 ± 1.32	4.79 ± 1.84
– dRNs	6.78 ± 1.62	5.54 ± 3.42
mRN		
– mRNa	9.67 ± 5.08	6.78 ± 2.28
– mRNs	10.02 ± 4.76	7.43 ± 1.72

*The BP_ND_ of the different volumes of interest in CD patients and controls. No significant differences were detected. Data are shown as mean ± SD. CD, cervical dystonia; BP_ND_, binding potential, non-displaceable; dRN, dorsal raphe nucleus, atlas based (dRNa) or subject based (dRNs); mRN, median raphe nucleus, atlas based (mRNa) or subject based (mRNs)*.

A trend with substantial effect size was detected toward decreased binding in the putamen of CD patients (9.3% decrease, *d* = 0.57, *p* = 0.09). Although the [^11^C]-DASB BP_ND_ was higher in the in the raphe nuclei of patients as compared with controls, this difference was not statistically significant in the dRN (dRNs 18.3% increase, *d* = 0.46, *p* = 0.21; dRNa 9.5% increase, *d* = 0.31, *p* = 0.22) nor in the mRN (mRNs 25.8% increase, *d* = 0.72, *p* = 0.16; mRNa 29.9% increase, *d* = 0.73, *p* = 0.45).

### Voxel-Based Analysis of [^11^C]DASB Binding

Similar to the VOI-based approach, the whole brain voxel-based analysis revealed no significant differences (*p* = 0.005, uncorrected) in the BP_ND_ between CD patients and controls.

### Correlations between Clinical Variables and [^11^C]DASB Binding

**Figure 2:**
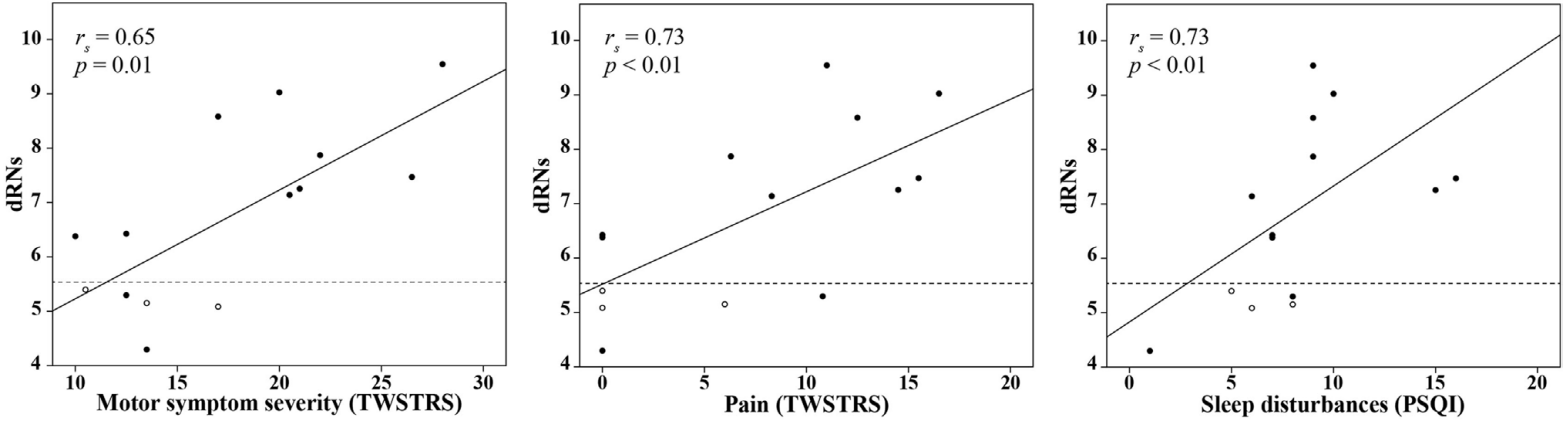
Correlation between the BP_ND_ in the dRNs in CD patients and clinical variables. Univariate correlation analysis in CD patients between the BP_ND_ in the dRNs and the TWSTRS motor severity score, TWSTRS pain score, and PSQI score. The dashed line indicates the mean BP_ND_ in the dRNs of the control group. Patients with the L_A_/L_A_ genotype are indicated as white circles. BP_ND_, binding potential, non-displaceable; dNRs: dorsal raphe nucleus, subject based; CD, cervical dystonia; *r*_s_, Spearman’s rho; TWSTRS, Toronto Western Spasmodic Torticollis Rating Scale; PSQI, Pittsburgh Sleep Quality Index.

In CD patients, motor symptom severity was significantly correlated with a higher BP_ND_ in the dRN (dRNs: *r*_s_ = 0.65, *p* = 0.01; dRNa: *r*_s_ = 0.60, *p* = 0.02). No other regions, particularly not the basal ganglia, showed such a correlation with motor symptom severity. Among the NMS scores, we found a significant correlation between the BP_ND_ in the dRN and pain (dRNs: *r*_s_ = 0.73, *p* < 0.01; dRNa: *r*_s_ = 0.58, *p* = 0.03) and sleep disturbances (dRNs: *r*_s_ = 0.73, *p* < 0.01, dRNa: *r*_s_ = 0.71, *p* < 0.01) (Figure 2). Patients with the L_A_/L_A_ genotype had a relatively low BP_ND_ in the dRNs, namely, 5.1, 5.2, and 5.4.

Subsequently, in the multiple regression analysis, we included motor symptom severity, scores on pain, and sleep disturbances to assess the effect on the BP_ND_ of the dRNs. As there was multicollinearity between pain and sleep disturbances scores (*r*_s_ = 0.87, *p* < 0.01), this was performed in two separate steps. First, we included motor severity and pain in the model. This revealed an adjusted *R*^2^ of 0.47; none of the variables significantly influenced the model. Second, we included motor symptom severity and sleep disturbances in the model. This revealed an adjusted *R*^2^ of 0.55, with motor symptom severity being the most important predictor of an increased BP_ND_ in the dRNs (β = 1.56, *p* = 0.02) (Table 3).

**Table 3:** Multiple regression analysis between the BP_ND_ of the dRNs and clinical variables.

Model	Region	Predictors	Adjusted *R*^2^	*B*°	β	*p*-Value
1	dRNs	– TWSTRS severity	0.47	0.10 (0.14)	0.34	0.49
		– TWSTRS pain		0.02 (0.22)	0.10	0.91
		– *Interaction effect*		0.00 (0.01)	0.38	0.75

2	dRNs	– TWSTRS severity	0.55	0.44 (0.16)	1.56	0.02
		– PSQI		0.70 (0.35)	1.65	0.07
		– *Interaction effect*		−0.03 (0.02)	−2.21	0.09

*Results of the multiple regression analysis to assess the effect of the TWSTRS severity score, the TWSTRS pain score, the PSQI score, and additional interaction effects on the BP_ND_ in the dRNs in CD patients. Due to multicollinearity between the TWSTRS pain score and PSQI score, we performed the analysis in two separate steps. For all variables, we calculated the *B*° (unstandardized coefficient with standard error in parenthesis) and β (standardized regression coefficient). BP_ND_, binding potential, non-displaceable; dRNs, dorsal raphe nucleus, subject based; TWSTRS, Toronto Western Spasmodic Torticollis Rating Scale; PSQI, Pittsburgh Sleep Quality Index*.

Furthermore, fatigue was negatively associated with the BP_ND_ in the mRN (mRNs: *r*_s_ = −0.61, *p* = 0.045, mRNa: *r*_s_ = −0.63, *p* = 0.049), while the domain sleep disorders was positively associated with the BP_ND_ in the caudate nucleus (*r*_s_ = 0.58, *p* = 0.03) and the hippocampus (*r*_s_ = 0.56, *p* = 0.02).

## Discussion

In this functional brain imaging study, we demonstrated, to the best of our knowledge for the first time, statistically significant positive correlations between [^11^C]DASB BP_ND_ in the dRN of CD patients and the severity of dystonic motor symptoms, pain scores, and sleep disturbances, but not with psychiatric comorbidity. Interestingly, the regression analysis showed that particularly the severity of the motor symptoms was the most important predictor of the model. Also, we found a trend toward a decrease of SERT binding in the putamen. Finally, scores of fatigue were negatively associated with the BP_ND_ in the mRN, while sleep disorder symptoms showed an association with BP_ND_ in the caudate nucleus and hippocampus.

The dRN and mRN are part of the eight raphe nuclei located within the brainstem. From these nuclei, serotonergic neurons project to several brain regions, including the basal ganglia, limbic region and cerebral cortex (20). SERT is highly prevalent both in the raphe nuclei and in the output areas, and forms an important presynaptic regulator of the serotonergic system. The highly presented SERT in regions involved in motor control (i.e., the basal ganglia) and in regions involved in NMS (i.e., the limbic region) and the functional interrelationship between these areas (21) suggest that alterations of SERT activity could contribute to the dystonia phenotype.

Increased SERT binding in the raphe nuclei related to motor and NMS in our cohort of CD patients might suggest an increased serotonergic state in CD, although causal relationships could not be extracted from this. The increased SERT binding could be explained by an upregulation due to an increased level of serotonin in the synaptic cleft. An increased serotonergic state related to dystonia is supported by many case reports of dystonia induced by selective serotonin reuptake inhibitors, but also by ondansetron, a potent 5-HT_3_ receptor antagonist [for review see Ref. (2)]. Further evidence of an increased serotonergic activity came from animal studies and studies describing other hyperkinetic extrapyramidal disorders. In both cat and monkey, serotonin injections in the facial nucleus induced focal dystonia of the eyelids (22). Also, administering the precursor of serotonin elicits a consistent hyperkinetic motor syndrome including lateral head waving (23). At the spinal cord level, serotonin increases spinal motor neuron excitability, facilitating the generation of action potentials. Dyskinesia is another condition of increased motor activity, occurring in Parkinson’s disease and it is associated with an increased ratio of SERT vs. dopamine transporter binding (7).

Opposite to the increased binding in the raphe nuclei, we found a trend toward decreased SERT binding in the putamen. One might argue that increased serotonergic activity in the raphe nuclei may increase 5-HT_1A_ autoreceptor activation within the same area, which results in an inhibitory effect on discharge patterns to the output areas, including the putamen (21). Interestingly, in Parkinson’s disease such reduced serotonergic activity in the basal ganglia by stimulating inhibitory 5-HT_1A_ receptors may ameliorate dyskinesia (7).

Not only motor severity but also pain and sleep disturbances showed a positive correlation with the BP_ND_ in the dRN. Moreover, sleep disturbance symptoms were also associated with SERT binding in the caudate nucleus and hippocampus. Serotonin involvement in pain is supported by previous studies. For example, spinal analgesic action is mediated by serotonin release, and selective serotonin reuptake inhibitors induce a central analgesic effect (24). In sleep disorders, altered discharge patterns of serotonergic neurons were suggested to be involved by influencing the different serotonergic postsynaptic receptors (25). Particularly, the REM sleep seems to be controlled by serotonergic activity. The REM sleep paralysis is mediated by inhibition of motoneurons, during which activity of serotonergic neurons is also suppressed. Moreover, inactivation of the dRN in animals caused a condition similar to REM sleep and simultaneously induced muscle paralysis (23). Altogether, the relation between SERT binding in the dRN and motor severity, pain, and sleep disturbances supports our hypothesis that serotonin forms a shared pathophysiological pathway of motor and NMS in dystonia.

For the interpretation of our results, it is important to keep in mind that the level of regionally measured [^11^C]DASB BP_ND_ reflects the *B*_max_/*K*_d_, or in other words the number of SERTs, and the affinity of the radiotracer for the SERT, which may be influenced by the quantity of endogenous synaptic serotonin, competing at the binding sites. For example, an increase of endogenous serotonin may reduce [^11^C]DASB binding without a change in the actual number of binding sites. The latter has particularly been demonstrated in acute experimental conditions of drugs employed to manipulate serotonin availability (26). However, in chronic conditions of low-serotonin concentrations, a downregulation of SERT is thought to occur to preserve synaptic 5-HT levels. Thus, lower binding in the basal ganglia output regions may be explained by several mechanisms, and the mechanism involved in our study is as yet unclear. Although the patients included in our study were characterized by a distinct profile of symptoms, the finding of a significant correlation between symptom severity and increased dRN [^11^C]DASB binding provides a strong argument to regard this dRN binding as a fair indicator of increased presynaptic serotonergic activity.

This study has some limitations. Scans were performed on two different cameras. However, both cameras are in the same center, are almost similar, and use the same reconstruction protocol. We did not include age and gender as covariates. As participants were matched on age and gender, we do not believe this decision significantly influenced the results. Side-related correlations between SERT binding and clinical characteristics (e.g., rotation, lateroflexion) could unfortunately not be answered based on 14 participating patients. Another limitation is that motor scores were obtained during the period with the least influence of botulinum toxin, while the scans were performed in the period of the maximum botulinum toxin effect. Therefore, the different SERT binding between CD patients and controls might even be an underestimation, caused by the therapeutic effect of botulinum toxin. Another consideration is the effect of BoNT on SERT binding. While BoNT may enter the spinal cord, a neurochemical interaction with brainstem serotonin neurotransmission does not seem plausible. On the other hand, the BoNT effect of (temporary) dystonic relief has indeed been described to induce altered functional network organization. For example, functional imaging studies (27, 28) have shown that BoNT therapy was associated with an increase in BOLD response in particularly somatosensory-related brain regions. The methodology underlying such functional imaging studies, including repeated within subject measurements to achieve statistical power, enables identification of subtle changes in responses. PET imaging with SERT implies investigating the more basal cerebral alterations of a neurotransmitter system at a steady-state condition. For this reason, we do not assume that BoNT had an effect on tracer uptake in the PET method.

Another limitation concerns the relationship between NMS and SERT binding in CD patients, while we could not examine this possible relation in the control group as those symptoms were lacking. In the future, it would be interesting to examine a possible relation between those symptoms and SERT binding independently from the dystonia pathophysiology, which would enable to discriminate between pathophysiology-related relationships or independent processes. At least, SERT binding in the raphe nuclei is challenging, due to the small size comprising only a couple of voxels. Therefore, we measured SERT binding in the dRN and mRN in two separate ways, namely, based on the MNI coordinates and visually by selecting the most active voxels. Because both techniques showed the same results, we do think we reliably measured SERT binding in the raphe nuclei.

In conclusion, this study provides the first evidence of the involvement of the serotonergic system in both motor and NMS in CD, which motivates the design of future studies focusing on this neurotransmitter system, including imaging studies on serotonin receptors. Eventually, such an approach may pave the way to evaluate the effect of serotonergic drugs on the motor and NMS symptoms in CD.

## Ethics Statement

Informed consent was obtained from all participants in accordance with the declaration of Helsinki and the study was approved by the local ethics committee of the University Medical Center Groningen (2014/034).

## Author Contributions

MS: design, acquisition, analysis, interpretation, drafting, approval, and agreement. DG: acquisition, analysis, interpretation, drafting, approval, and agreement. BJ: acquisition, analysis, interpretation, drafting and revising, approval, and agreement. EZ, JB, and RD: design, interpretation, revising, approval, and agreement. AW and EV: conception, design, interpretation, revising, approval, and agreement. AB: conception, design, acquisition, analysis, interpretation, drafting, approval, and agreement. MT: design, interpretation, revising, approval, and agreement.

## Conflict of Interest Statement

MS has received grants from the University Medical Centre Groningen and Stichting Wetenschapsfonds Dystonie Vereniging. MT has received research grants from Stichting Wetenschapsfonds Dystonie Vereniging, Prinses Beatrix Foundation, Fonds NutsOhra, and unrestricted grants for DystonieNet from Ipsen Pharmaceuticals, Allergan Pharmaceuticals, Medtronic, and Actelion. The study was supported by the University Medical Center Groningen—Healthy Ageing Pilot Fund.

## References

[1] Klingelhoefer L Martinoa D Martinez-Martinc P Sauerbiera A Rizosa A Jostd W Non-motor symptoms and focal cervical dystonia: observations from 102 patients. Basal Ganglia (2014) 4(3–4):117–20. 10.1016/j.baga.2014.10.002

[2] Smit M Kuiper A Han V Jiawan VC Douma G van Harten B Psychiatric co-morbidity is highly prevalent in idiopathic cervical dystonia and significantly influences health-related quality of life: results of a controlled study. Parkinsonism Relat Disord (2016) 30:7–12. 10.1016/j.parkreldis.2016.06.004 27321988

[3] Vidailhet M Jutras MF Grabli D Roze E. Deep brain stimulation for dystonia. J Neurol Neurosurg Psychiatry (2013) 84(9):1029–42. 10.1136/jnnp-2011-301714 23154125

[4] Quartarone A Hallett M. Emerging concepts in the physiological basis of dystonia. Mov Disord (2013) 28(7):958–67. 10.1002/mds.25532 23893452 PMC4159671

[5] Smit M Bartels AL van Faassen M Kuiper A Niezen-Koning KE Kema IP Serotonergic perturbations in dystonia disorders-a systematic review. Neurosci Biobehav Rev (2016) 65:264–75. 10.1016/j.neubiorev.2016.03.015 27073048

[6] Loane C Wu K Bain P Brooks DJ Piccini P Politis M. Serotonergic loss in motor circuitries correlates with severity of action-postural tremor in PD. Neurology (2013) 80(20):1850–5. 10.1212/WNL.0b013e318292a31d 23596065 PMC3908354

[7] Lee JY Seo S Lee JS Kim HJ Kim YK Jeon BS. Putaminal serotonergic innervation: monitoring dyskinesia risk in Parkinson disease. Neurology (2015) 85(10):853–60. 10.1212/WNL.0000000000001909 26253444

[8] Pavese N Metta V Bose SK Chaudhuri KR Brooks DJ Fatigue in Parkinson’s disease is linked to striatal and limbic serotonergic dysfunction. Brain (2010) 133(11):3434–43. 10.1093/brain/awq268 20884645

[9] Politis M Wu K Loane C Turkheimer FE Molloy S Brooks DJ Depressive symptoms in PD correlate with higher 5-HTT binding in raphe and limbic structures. Neurology (2010) 75(21):1920–7. 10.1212/WNL.0b013e3181feb2ab 21098407

[10] Consky ES Lang AE Clinical assessments of patients with cervical dystonia. In: Jankovic J Hallett M, editors. Therapy with Botulinum Toxin. New York, NY: Marcel Dekker (1994). p. 211–37.

[11] Guy W The clinical global impression scale. ECDEU Assessment Manual for Psychopharmacology-Revised. Rockville, MD: US Dept. of Health, Education and Welfare, ADAMHA, NIMH Psychopharmacology Research Branch (1976). 218 p.

[12] Hu XZ Lipsky RH Zhu G Akhtar LA Taubman J Greenberg BD Serotonin transporter promoter gain-of-function genotypes are linked to obsessive-compulsive disorder. Am J Hum Genet (2006) 78(5):815–26. 10.1086/503850 16642437 PMC1474042

[13] Wilson AA Ginovart N Schmidt M Meyer JH Threlkeld PG Houle S. Novel radiotracers for imaging the serotonin transporter by positron emission tomography: synthesis, radiosynthesis, and in vitro and ex vivo evaluation of (11)C-labeled 2-(phenylthio)araalkylamines. J Med Chem (2000) 43(16):3103–10. 10.1021/jm000079i 10956218

[14] Ashburner J Friston KJ. Unified segmentation. Neuroimage (2005) 26(3):839–51. 10.1016/j.neuroimage.2005.02.018 15955494

[15] Hammers A Allom R Koepp MJ Free SL Myers R Lemieux L Three-dimensional maximum probability atlas of the human brain, with particular reference to the temporal lobe. Hum Brain Mapp (2003) 19(4):224–47. 10.1002/hbm.10123 12874777 PMC6871794

[16] Kranz GS Hahn A Savli M Lanzenberger R Challenges in the differentiation of midbrain raphe nuclei in neuroimaging research. Proc Natl Acad Sci U S A (2012) 109(29):E2000 10.1073/pnas.1206247109 22711836 PMC3406805

[17] Innis RB Cunningham VJ Delforge J Fujita M Gjedde A Gunn RN Consensus nomenclature for in vivo imaging of reversibly binding radioligands. J Cerebral Blood Flow Metab (2007) 27(9):1533–9. 10.1038/sj.jcbfm.9600493 17519979

[18] Wu Y Carson RE. Noise reduction in the simplified reference tissue model for neuroreceptor functional imaging. J Cerebral Blood Flow Metab (2002) 22(12):1440–52. 10.1097/01.WCB.0000033967.83623.34 12468889

[19] Field A Discovering Statistics Using SPSS. Thousand Oaks, California: SAGE (2009).

[20] Ohno Y Shimizu S Tokudome K. Pathophysiological roles of serotonergic system in regulating extrapyramidal motor functions. Biol Pharm Bull (2013) 36(9):1396–400. 10.1248/bpb.b13-00310 23995648

[21] Sibille E Lewis DA SERT-ainly involved in depression, but when? Am J Psychiatry (2006) 163:8 10.1176/appi.ajp.163.1.8 16390880

[22] LeDoux MS Lorden JF Smith JM Mays LE. Serotonergic modulation of eye blinks in cat and monkey. Neurosci Lett (1998) 253(1):61–4. 10.1016/S0304-3940(98)00616-8 9754805

[23] Jacobs BL Fornal CA. 5-HT and motor control: a hypothesis. Trends Neurosci (1993) 16(9):346–52. 10.1016/0166-2236(93)90090-9 7694403

[24] Sommer C. Serotonin in pain and analgesia: actions in the periphery. Mol Neurobiol (2004) 30(2):117–25. 10.1385/MN:30:2:117 15475622

[25] Portas CM Bjorvatn B Ursin R. Serotonin and the sleep/wake cycle: special emphasis on microdialysis studies. Prog Neurobiol (2000) 60(1):13–35. 10.1016/S0301-0082(98)00097-5 10622375

[26] Yamamoto S Onoe H Tsukada H Watanabe Y Effects of increased endogenous serotonin on the in vivo binding of [11C]DASB to serotonin transporters in conscious monkey brain. Synapse (2007) 61(9):724–31. 10.1002/syn.20422 17559093

[27] Delnooz CC Pasman JW van de Warrenburg BP. Dynamic cortical gray matter volume changes after botulinum toxin in cervical dystonia. Neurobiol Dis (2015) 73:327–33. 10.1016/j.nbd.2014.10.013 25447226

[28] Opavsky R Hluštík P Otruba P Kaňovský P Somatosensory cortical activation in cervical dystonia and its modulation with botulinum toxin: a fMRI study. Int J Neurosci (2012) 122(1):45–51. 10.3109/00207454.2011.623807 21919815

