# Supplementary material for: Relationships between Serotonin Transporter Binding in the Raphe Nuclei, Basal Ganglia, and Hippocampus with Clinical Symptoms in Cervical Dystonia: A [11C]DASB Positron Emission Tomography Study
Source: Front Neurol. 2018 Feb 28;9:88. doi: 10.3389/fneur.2018.00088 (PMC5835525; doi:10.3389/fneur.2018.00088)
Supplement: Supplementary file 1 [file data_sheet_1.docx]

**Supplementary text: SERT genotyping**

The SERT S/L_A_/L_G_ variants were determined using polymerase chain reaction (PCR) with Forward primer FAM-5'TGAATGCCAGCACCTAACCC-3' and Reverse primer 5-TTCTGGTGCCACCTAGACGC-3', and subsequent ingestion of the PCR product with Msp-I for at least 3 hours at 37 °C. The resulting restriction fragments were separated using capillary electrophoresis (ABI 3130 analyzer; Applied Biosystems, the Netherlands) and fragment sizes were estimated using the ABI Prism® GeneMapper™ software, version 3.0 (Applied Biosystems). The S, L_G_ and L_A_ variants were determined by detection of fragments of 325, 152 or 284 base pairs, respectively.
